# Antimicrobial resistance and virulence signatures of *Listeria* and *Aeromonas* species recovered from treated wastewater effluent and receiving surface water in Durban, South Africa

**DOI:** 10.1186/s12866-015-0570-x

**Published:** 2015-10-24

**Authors:** Ademola O. Olaniran, Sphephile B T Nzimande, Ndumiso G. Mkize

**Affiliations:** Discipline of Microbiology, School of Life Sciences, College of Agriculture, Engineering and Science, University of KwaZulu-Natal (Westville Campus), Private Bag X54001, Durban, 4000 Republic of South Africa

**Keywords:** *Listeria* spp, *Aeromonas* spp, Virulence genes, Antibiotic resistance, Wastewater effluent

## Abstract

**Background:**

Treated wastewater effluent has been found to contain high levels of contaminants, including disease-causing bacteria such as *Listeria* and *Aeromonas* species. The aim of this study was to evaluate the antimicrobial resistance and virulence signatures of *Listeria* and *Aeromonas* spp. recovered from treated effluents of two wastewater treatment plants and receiving rivers in Durban, South Africa.

**Methods:**

A total of 100 Aeromonas spp. and 78 Listeria spp. were positively identified based on biochemical tests and PCR detection of DNA region conserved in these genera. The antimicrobial resistance profiles of the isolates were determined using Kirby Bauer disc diffusion assay. The presence of important virulence genes were detected via PCR, while other virulence determinants; protease, gelatinase and haemolysin were detected using standard assays.

**Results:**

Highest resistance was observed against penicillin, erythromycin and nalidixic acid, with all 78 (100 %) tested *Listeria* spp displaying resistance, followed by ampicillin (83.33 %), trimethoprim (67.95 %), nitrofurantoin (64.10 %) and cephalosporin (60.26 %). Among *Aeromonas* spp., the highest resistance (100 %) was observed against ampicillin, penicillin, vancomycin, clindamycin and fusidic acid, followed by cephalosporin (82 %), and erythromycin (58 %), with 56 % of the isolates found to be resistant to naladixic acid and trimethoprim. Among *Listeria* spp., 26.92 % were found to contain virulence genes, with 14.10, 5.12 and 21 % harbouring the *act*A, *plc*A and *iap* genes, respectively. Of the 100 tested *Aeromonas* spp., 52 % harboured the aerolysin (*aer*) virulence associated gene, while lipase (*lip*) virulence associated gene was also detected in 68 % of the tested *Aeromonas* spp.

**Conclusions:**

The presence of these organisms in effluents samples following conventional wastewater treatment is worrisome as this could lead to major environmental and human health problems. This emphasizes the need for constant evaluation of the wastewater treatment effluents to ensure compliance to set guidelines.

## Background

Wastewater effluent and surrounding fresh water bodies such as rivers and estuaries have been found to contain high levels of contaminants, including disease-causing bacteria such as *Listeria* spp. and *Aeromonas* spp. [1]. The ability of these organisms to survive conventional wastewater treatment processes could lead to major environmental and human health problems, resulting from the highly contaminated surface waters [1]. Previously, *Listeria* has only been associated with food related infections and diseases, but has now been discovered and reported in water [2]. Of the seven recognised *Listeria* spp. (*L. monocytogenes, L. ivanovii. L. innocua, L. seeligeri, L. welshimeri, L. grayii* and *L. murrayi*), only *L. monocytogenes* and *L. ivanovii* are currently deemed as pathogenic and infectious, causing diseases in animals and human beings, since they are known to display β-haemolytic activity [3–6]. *Listeria* spp., mainly *L. monocytogenes* cause listeriosis which develops mostly in neonates, the elderly, pregnant women, and immuno-compromised individuals [2, 7]. Infection by *L. monocytogenes* occurs in several steps, each requiring expression of specific virulence factor. The major virulence genes are located in a cluster of genes on two different DNA loci and are mainly influenced by positive regulatory factor A protein. Important virulence factors have been characterised; listeriolysin O encoded by the gene *hlyA* and phosphatidylinositol-specific phospholipase C encoded by the gene *plcA*. These genes play an important role in lysis of the phagosomes of the host cell and this makes the intracellular growth of *Listeria* cells possible [8, 9].

Aeromonads have grown in importance as food and waterborne pathogens, receiving much research attention since their discovery and implication in gastrointestinal diseases [10–12]. Seven *Aeromonas* spp., (A*. hydrophila, A. caviae, A. veronii biovar sobria, A. veronii biovar veronii, A. jandaei, A. trota,* and *A. schubertii*) are currently recognized as human pathogens [13]. Aeromonads are mesophilic bacteria which are found and widely distributed in soil and mostly aquatic (fresh, marine, estuarian) environments. They are mostly opportunistic microorganisms, mainly affecting individuals with reduced or compromised immunity, children and the elderly [14]. *Aeromonas* spp., have been widely reported in diarrheal diseases based on their common discovery in faecal samples of patients suffering from diarrhoea and other gastrointestinal diseases [14, 15]. The wide presence of potentially pathogenic *Aeromonas* spp. in freshwater bodies is of major public health concern [16]. A variety of potential virulence factors and toxins have been characterised in *Aeromonas* spp. [17], the most common ones being *aer* and *hlyA* gene which are responsible for aerolysin and hemolysin toxins production, respectively [18]. Aerolysin is one of the major virulence factors in gastroenteritis and in invasion of epithelial cells [19]. Some studies have reported high antimicrobial resistance patterns in *Aeromonas* spp., which increases the public threat, especially in cases of immunocompromised individuals with severe infections [16].

Wastewater treatment plants across South Africa have displayed poor bacterial pathogen removal over the years [1]. Furthermore, the current treatment regulations and guidelines mainly using common indicator organisms as a standard for monitoring drinking water and wastewater treatment processes have proven to be unreliable [20, 21]. This is of great public concern considering that an estimated 77 % of South Africans depend on surface water for most of their domestic water needs [22, 23]. Worse cases of waterborne infections have been seen and reported mostly in poverty stricken populations which lack adequate sanitation and infrastructure as surrounding populations have easy and uncontrolled access to free-flowing, highly contaminated waters to meet their water needs [1]. The prevalence of *Listeria* and *Aeromonas* spp. in treated wastewater effluent has been reported both in developing and developed countries [1, 2, 24]. Also, wastewater has been reported to be a potential reservoir and transporter of pathogenic *Listeria* and *Aeromonas* which harbour virulence determinants and display a notable trend of resistance to commonly used antimicrobial agents [2, 7, 24]. However, there is no report on the prevalence, antibiogram and virulence signatures of *Listeria* and *Aeromonas* spp. in effluent discharge of wastewater treatment plants in KwaZulu-Natal province of South Africa. This study therefore evaluated the antibiotic resistance phenotype and virulence properties of *Listeria* and *Aeromonas* spp. recovered from treated effluent of two wastewater treatment plants in Durban.

## Methods

### Water sampling and bacterial isolation

Wastewater and river samples were collected from two different wastewater treatment plants, designated as NWTW and NGWTW, located in the Durban area (KwaZulu-Natal, South Africa). Permissions were obtained from the relevant authority of both treatment plants to carry out water sampling. Water samples were collected before and after chlorination of the final treated effluent, and approximately 500 m up and downstream of discharge point into the receiving waters. Samples were serially diluted with sterile distilled water and 50 ml of the appropriate dilutions were filtered using standard 0.45 μm pore sized filters, according to standard membrane filtration methods. Membrane filters were then aseptically transferred onto Rimler-shotts agar (HIMEDIA, India) (incubated at 37 °C for 20 h) and *Listeria* Chromogenic agar (Oxoid, Cambridge, UK) (incubated at 35 °C for 24–48 h) for identification of *Aeromonas* spp and *Listeria* spp., respectively. Presumptive isolates with typical appearance on the respective medium (yellow for *Aeromonas* and blue for *Listeria*) were inoculated separately onto fresh selective media to obtain pure culture, before sub-culturing onto nutrient agar plates for identification.

### Identification of the bacterial isolates

#### Biochemical identification of *Listeria and Aeromonas* spp.

Identification of the presumptive *Aeromonas* and *Listeria* spp. recovered from treated effluent and the receiving rivers was carried out using a range of biochemical tests, including oxidase, catalase, urease, carbohydrate fermentation, acid formation (TSI), indole production tests, Methyl red and Voges-Proskauer test [25, 26].

#### Molecular identification

Polymerase chain reaction (PCR) was used to amplify and detect the presence of specific conserved sequences in presumptive *Listeria* spp. and *Aeromonas* spp. isolates using primers List-universal 1 and 2, and gyrB3F and gyrB14R, respectively (Inqaba Biotech, SA) (Table 1). DNA was extracted using the boiling method according to Bai et al. modified protocol [27]. All PCR amplifications were performed in a thermal cycler (GeneAMP PCR System 2400, Bio-Rad). For the detection of *iap* gene in *Listeria* spp., PCR conditions were as follows: Pre-denaturation at 95 °C for 5 min, 30 cycles of; denaturation at 95 °C for 1 min, annealing at 36 °C for 1 min, extension at 72 °C for 3 min and final extension at 72 °C for 7 min [28]. PCR was performed in a final volume of 50 μl containing 50 mM KCl, 10 mM Tris/HCl (pH 8.3), 3.5 mM MgCl_2_, 0.2 μM of each dNTPs (Thermo Scientific Fermenters, UK), 1.25 U Supertherm *Taq* DNA polymerase (Separation Scientific, Cape Town, SA), 0.2 μM of each primer (Inqaba Biotech, SA) and 1 μl of the extracted DNA sample [28]. *Listeria monocytogenes* ATCC 19115 was used as a positive control in all PCR assays. For the PCR amplification of *gyrB* gene in *Aeromonas* spp., the following conditions were applied: Pre-denaturation at 94 °C for 2 min, 30 cycles of; denaturation at 93 °C for 30 s, annealing at 62 °C for 30 s and extension at 72 °C for 1 min [29]. PCR was performed in a final volume of 50 μl containing 50 mM KCl, 10 mM Tris/HCl (pH 9), 1.5 mM MgCl_2_, 0.2 mM dNTPs (Thermo Scientific Fermenters, UK), 1U Supertherm *Taq* DNA polymerase (Separation Scientific, Cape Town, SA), 20 pmol of each primer (Inqaba Biotech, SA) and 1 μl of the extracted DNA sample [30]. *Aeromonas caviae* ATCC 15468 and *Aeromonas hydrophila* ATCC 7965 strain were used as positive controls.

**Table 1:** Characteristics of primers used for PCR amplification of genus specific genes and virulence genes

Organism	Primer	Sequence (5′–3′)	Product size (bp)	References
*Listeria monocytogenes*	Plc A	CTG CTT GAG CGT TCA TGT CTC ATC CCC C	1484	[35]
		ATG GGT TTC ACT CTC CTT CTA C		
	Act A	CGC CGC GGA AAT TAA AAA AAG A	839	
		ACG AAG GAA CCG GGC TGC TAG		
	Iap	ACA AGC TGC ACC TGT TGC AG	131	
		TGA CAG CGT GTG TAG TAG CA		
*Aeromonas spp.*	aer-F	CCTATGGCCTGAGCGAGAAG	431	[36]
	aer-R	CCAGTTCCAGTCCCACCACT		
	lip-F	CA(C/T)CTGGT(T/G)CCGCTCAAG	247	
	lip-R	GT(A/G)CCGAACCAGTCGGAGAA		
*Listeria* spp.	List-Universal 1	ATGTCATGGAATAA	457–610	[28]
	List-Universal 2	GCTTTTCCAAGGTGTTTTT		
*Aeromonas* spp.	gyrB3F	TCCGGCGGTCTGCACGGCGT	1100	[29]
	gyrB14R	TTGTCCGGGTTGTACTCGTC		

### Antimicrobial susceptibility determination

The Kirby Bauer Disk diffusion method [31, 32] was used to determine the antibiotic resistance profile of *Listeria* and *Aeromonas* spp. isolates. The isolates were screened against a panel of 24 antibiotics belonging to 14 different classes (Table 2). Cultures were grown for 24 h in Luria-Bertani broth and thereafter standardized to 0.5 McFarland standard (OD_625nm_ = 0.08–0.1) using a spectrophotometer (Biochrom, Libras), before spreading on Mueller-Hinton agar plates using sterile swabs. The plates were then dried at room temperature for 15 min before placing 4 discs per plates at approximately 40 mm apart from each other. The plates were incubated for 18–24 h at 35–37 °C. Zones of clearance surrounding each disk were used to determine the level of susceptibility or resistance, and were scored based on the CLSI standards [31, 32] using *Escherichia coli* ATCC 25922 as standard for Gram negative and *Staphylococcus aureus* ATCC 25923 as a standard for Gram positive [33]. Multiple Antibiotic Resistance (MAR) index was calculated as follows: MAR = a/b, where a = number of antibiotics to which the isolate was resistant; b = total number of antibiotics against which individual isolate was tested.

**Table 2:** Antimicrobial resistance/susceptibility profile of *Aeromonas* and *Listeria* species isolates

Antibiotic class	Antibiotics	Conc (μg)	Bacterial isolates					
			*Aeromonas* spp. (*n* = 100)			*Listeria spp. (n = 78)*		
			n (Resistant)	n (Susceptible)	n (Intermediate)	n (Resistant)	n (Susceptible)	n (Intermediate)
β-Lactams	Penicillin (P)	10 (10)	100 (100)	0 (0)	0 (0)	78 (100)	0 (0)	0 (0)
	Cephalothin (KF)	30	82 (82)	15 (15)	3 (3)	47 (60.26)	24 (30.77)	7 (8.97)
Aminoglycosides	Gentamicin (CN)	10	0 (0)	100 (100)	0 (0)	0	72 (92.31)	6 (7.69)
	Kanamycin (K)	5	14 (14)	59 (59)	27 (27)	41 (52.56)	18 (23.08)	19 (24.36)
	Amikacin (AK)	30	0 (0)	95 (95)	5 (5)	0	78 (100)	0
Carbapenems	Ertapenem (ETP)	10	23 (23)	72 (72)	5 (5)	22 (28.21)	48 (61.54)	8 (10.25)
	Meropenem (MEM)	10	11 (11)	79 (79)	10 (10)	0 (0)	70 (89.74)	8 (10.26)
Cephalosporin	Cefotaxime (CTX)	30	6 (6)	82 (82)	12 (12)	24 (30.77)	39 (50)	15 (19.23)
	Ceftriaxone (CRO)	30	22 (22)	78 (78)	0 (0)	19 (24.36)	45 (59.69)	14 (17.95)
Glycopeptides	Vancomycin (VA)	30	100 (100)	0 (0)	0 (0)	24 (30.77)	54 (69.23)	0 (0)
Lincosamides	Clindamycin (DA)	10	100 (100)	0 (0)	0 (0)	9 (11.54)	69 (88.46)	0 (0)
Macrolides	Erythromycin (E)	15	58 (58)	11 (11)	31 (31)	78 (100)	0 (0)	0 (0)
Nitrofurans	Nitrofurantoin (F)	50	4 (4)	72 (72)	24 (24)	50 (64.10)	6 (7.69)	22 (28.20)
Penicillins	Ampicillin (AMP)	10	100 (100)	0 (0)	0 (0)	65 (83.33)	8 (10.26)	5 (6.41)
Polypeptides	Colistin (CT)	10	14 (14)	86 (86)	0 (0)	16 (20.51)	62 (79.48)	0 (0)
Quinolones	Nalidixic acid (NA)	30	56 (56)	44 (44)	0 (0)	78 (100)	0 (0)	0 (0)
	Mixofloxacin (MXF)	5	3 (3)	83 (83)	14 (14)	3 (3.85)	72 (92.30)	3 (3.85)
	Ciprofloxacin (CIP)	5	0 (0)	94 (94)	6 (6)	0	75 (96.15)	3 (3.85)
Sulfonamides	Trimethoprim (W)	5	56 (56)	38 (38)	6 (6)	53 (67.95)	20 (25.64)	5 (6.41)
Tetracyclines	Tetracycline (TE)	10	19 (19)	44 (44)	37 (37)	37 (47.44)	31 (39.74)	10 (12.82)
Other	Streptomycin (S)	25	16 (16)	70 (70)	14 (14)	0	78 (100)	0
	Chloramphenicol (C)	30	4 (4)	95 (95)	1 (1)	0	78 (100)	0
	Fosfomycin (FOS)	50	3 (3)	88 (88)	9 (9)	0	78 (100)	0
	Fusidic Acid (FD)	10	100 (100)	0 (0)	0 (0)	0	78 (100)	0

### Protease, gelatinase and haemolysin assay

Protease activity was assayed by spreading bacterial strains on nutrient agar containing 1.5 % skim milk. After incubation at 30 °C for 72 h, the production of protease was shown by the formation of a clear zone caused by casein degradation. Gelatinase production was determined on LB agar containing gelatine (30 g/L). The plates were incubated at 30 °C for 24 h and cooled for 5 h at 4 °C. The appearance of turbid halos around the colonies was considered positive for gelatinase production [34]. Haemolysin production was assayed by culturing each strain on blood agar at 30 °C for 24 h. The production of haemolysin was observed by the formation of a clear zone caused by β-haemolysis activity of the enzyme on the blood (modified from Sechi et al. [35].

### PCR detection of virulence genes

A multiplex PCR assay was used for the detection of four virulence-associated genes of *L. monocytogenes* namely, *plcA*, *hlyA*, *actA* and *iap*, coding for the phospholipase, haemolysin, intracellular motility and p60 invasion proteins, respectively [35] using the primer (Inqaba Biotech, SA) sets indicated in Table 1. The final reaction mixture (50 μl) contained: 1 × PCR buffer, 1 mM dNTP mix (Thermo Scientific Fermenters, UK), 6 mM MgCl_2_ and 10 μM of each primer sets, 4U of Supertherm *Taq* DNA polymerase (Separation Scientific, Cape Town, SA), 5 μl of DNA and sterilized water to make up the reaction volume. PCR was carried out in a Thermocycler (GeneAMP PCR System 2400, Bio Rad) under the conditions stated by Rawool et al. [35]. A monoplex PCR was used for the detection of two virulence-associated genes of *Aeromonas* spp. namely, *aer* and *lip*, coding for the aerolysin and lipase enzymes, respectively, using the primer sets (Inqaba Biotech, SA) indicated in Table 1. Each reaction was carried out in a total volume of 25 μl, containing 12.5 μl of the PROMEGA G2 Go *Taq* green master mix (ANATECH), 5 μl of isolated genomic DNA and sterile double distilled water to make up the reaction volume. PCR was carried out in a Thermocycler (GeneAMP PCR System 2400, Bio Rad) under the conditions stated by Igbinosa et al. [36].

## Results

### Identification of the presumptive Aeromonas and Listeria spp. isolates

**Fig. 1:**
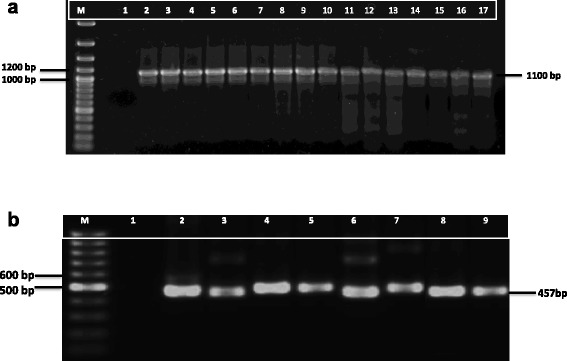
**a**. Agarose gel showing PCR amplicons of the *gyrB* gene of representative *Aeromonas* spp. isolates (lanes 2–17), M: 100 pb molecular marker and lane 1: negative control. **b**. Agarose gel showing PCR amplicons of the *iap* gene of representative *Listeria* spp. isolates (lanes 2–9), M: molecular marker and lane 1: negative control

*Aeromonas* spp. isolates were confirmed as either negative or positive for the biochemical tests conducted. Oxidase and catalase positive, urease negative, Methyl red positive and Voges-Proskauer negative organisms were confirmed as *Aeromonas* spp. The *gyrB* gene region was successfully amplified in positive isolates with the expected product size (1100 bp), as shown in Fig. 1a. Biochemical reaction of *Listeria* isolates was shown by oxidase negative, catalase positive and Methyl red and Voges-Proskauer positive. All positively identified *Listeria* spp. isolates were further confirmed by PCR, with the expected amplicon sizes (457–610 bp, commonly 457 bp) of the universal conserved *iap* gene obtained (Fig. 1b).

### Antimicrobial resistance profiles of Listeria and Aeromonas spp. isolates

**Fig. 2:**
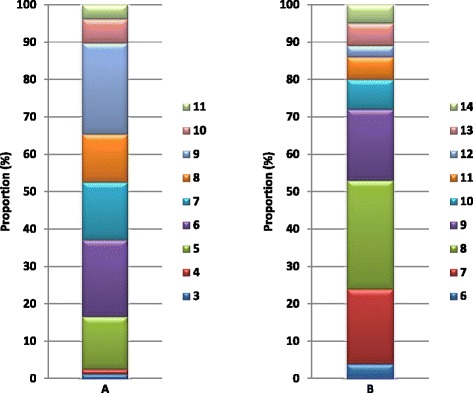
Multidrug resistance patterns in (**a**) *Listeria* spp. and (**b**) *Aeromonas* spp. indicating the percentage isolates resistant to different number of antibiotic classes

The resistance and susceptibility profiles of *Listeria* spp. and *Aeromonas* spp. isolates against a broad range of antimicrobials commonly used for Enterobacteria are shown in Table 2. Among *Listeria* spp., the highest resistance (100 %) was observed against penicillin, erythromycin and nalidixic acid, followed by ampicillin (83.33 %), trimethoprim (67.95 %), nitrofurantoin (64.10 %) and cephalosporin (60.26 %). Of the 78 tested *Listeria* spp. isolates, all (100 %) were found to be sensitive to 5 of the antibiotics: streptomycin, chloramphenicol, fosfomycin, fusidic acid and amikacin, followed by ciprofloxacin (96.15 %), gentamicin (92.31 %), mixofloxacin (92.31 %), meropenem (89.74 %), clindamycin (88.46 %) and colistin (79.48 %). All the tested isolates showed resistance to at least 5 of the 24 antibiotics, with 4 (5.13 %) of the test isolates displaying resistance to at most 12 of the 24 antibiotics as shown in Table 2. The antibiotic resistance index (ARI) for the *Listeria* spp. ranged between 0.13 (resistance to 3 test antibiotics) and 0.5 (resistance to 12 of the test antibiotics). The multidrug resistance patterns of the *Listeria* spp. as shown in Fig. 2a, revealed that at least 1 (1.28 %) of the isolates was resistant to 3–4 antibiotic classes, with most of the multidrug resistant isolates being resistant to at least more than 5 and at most 11 antimicrobial classes. The highest multidrug resistance patterns was observed in 3 (3.85 %) of the 78 tested *Listeria* spp isolates, being resistant to 11 of the 24 tested antibiotics, while the highest percentage resistance (to 9 antimicrobial classes) was observed in 19 (24.36 %) isolates (Fig. 2a).

The resistance and susceptibility profiles of the 100 *Aeromonas* spp. isolates tested against a broad range of antimicrobials commonly used for the treatment of Enterobacteria-associated infections are shown in Table 2. The highest resistance (100 %) was observed for ampicillin, penicillin, vancomycin, clindamycin and fusidic acid, followed by cephalothin (82 %), and erythromycin (58 %), with 56 % of the isolates found to be resistant to nalidixic acid and trimethoprim. All the isolates were found to be sensitive to gentamycin, 95 % to amikacin and chloramphenicol, while 94, 88, 86, 83 and 82 % of the isolates were susceptible to ciprofloxacin, fosfomycin, colistin, mixofloxacin and cefotaxime, respectively. All the tested isolates showed resistance to at least 6 of the 24 antibiotics, with 5 of the test isolates displaying resistance to 14 of the 24 antibiotics as shown in Table 2. The multidrug resistance patterns of *Aeromonas* spp. displayed in Fig. 2b), show that most of the multidrug resistant isolates were resistant to at least 6 and at most 14 classes of antimicrobials, with the ARI ranging from 0.25 to 0.58. The highest multidrug resistance pattern (to 14 antimicrobial classes) was observed in 5 % of the test isolates, while 29 % of the tested isolates were resistant to 8 antimicrobial classes (Fig. 2b).

### Virulence gene signatures of the Listeria and Aeromonas spp. isolates

**Fig. 3:**
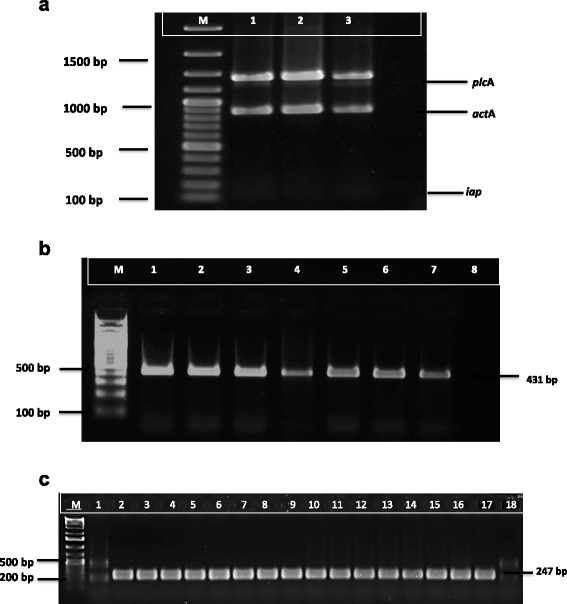
**a**. Agarose gel showing PCR amplicons of three virulence associated genes (*plcA*, *actA*, and *iap*) detected in representative *Listeria spp.* (lanes 1 & 3) and *L. monocytogenes* (ATCC 19115) (Lane 2). M: DNA marker (100 to 3000 bp) and Lane 4: negative control. **b**. Agarose gel showing PCR amplicons of the Aerolysin (*aer*) virulence associated gene of representative *Aeromonas* spp*.,* M: DNA marker (100 bp), lane 1–7: amplified PCR products, Lane 8: negative control. **c**. Agarose gel showing PCR amplicons of the Lipase (*lip*) virulence associated gene of representative *Aeromonas* spp*.,* M: DNA marker (100 bp), lane 1–17: amplified PCR products

The agarose gel showing the expected amplicon sizes of virulence associated gene products detected in some *Listeria* spp*.* isolates is represented in Fig. 3a. The primer sets allowed for the amplification of 1484 bp (*plc*A), 839 bp (*act*A) and 131 bp (*iap*) from the DNA template. Of the 78 tested *Listeria* spp., 21 (26.92 %) were found to contain virulence genes, with 14.10, 5.12 and 21 % of these species found to harbour *act*A, *plc*A and *iap* genes, respectively (Fig. 3a). Furthermore, 9 (11.54 %) of the isolates contained more than one virulence gene (*act*A and *iap* genes). PCR amplicons of the expected sizes of the *aer* gene (431 bp) and *lip* gene (247) in *Aeromonas* spp. is represented in Fig. 3b and c, respectively. Of the 100 tested *Aeromonas* spp., 52 % were found to harbour the *aer* virulence associated gene, while 68 % harboured *lip* virulence associated gene, with 29 isolates found to contain both the *aer* and *lip* genes.

### Protease, gelatinase and haemolysin production

Blood agar plates were hydrolysed by 25 (32 %) of the tested *Listeria* spp. by the formation of clear zones around the colonies indicating a positive result for the production of the haemolysin enzyme. However, all the *Listeria* isolates tested negative for gelatinase and protease enzyme production. All the 100 tested *Aeromomonas* spp. isolates tested positive for protease enzyme production, while 67 and 88 % of the isolates tested positive for the production of haemolysin and gelatinase, respectively.

## Discussion

The successful identification of the *Aeromonas* and *Listeria* spp using the biochemical and molecular methods indicates the prevalence of emerging bacterial pathogens in treated wastewater effluent, reiterating the fact that they are able to withstand and survive conventional wastewater treatment processes as previously reported [1, 13, 37, 38]. The observed highest resistance (100 %) of *Aeromonas* spp. against ampicillin, penicillin, vancomycin, clindamycin and fusidic acid in this study was in line with those reported in similar studies [13, 37, 39, 40], where ampicillin and vancomycin were amongst the antibiotics which had no antimicrobial activity towards tested *Aeromonas* spp. isolates. However, Goni-Urriza et al. [41] and P´erez-Valdespino et al. [42], did not observe complete resistance of *Aeromonas* spp. tested to these antibiotics, but a similar trend of high resistance levels was observed. Also, the high sensitivity patterns observed against gentamicin, fosfomycin, cefotaxime, amikacin, and meropenem in this study were comparable to previous findings [13, 39].

The susceptibility patterns of *Listeria* spp. obtained in this study are similar to those reported by Odjadjare and Okoh [1], who tested 23 *Listeria* isolates against 20 antibiotics and found that all tested isolates were sensitive to 3 of the 20 test antibiotics including amikacin (aminoglycosides), meropenem, and ertapenem (carbapenems) suggesting that these antibiotics may be the best therapy in the event of listeriosis outbreak in South Africa. In general, *L. monocytogenes*, as well as other strains of *Listeria* spp., are susceptible to a wide range of antibiotics [43], however a notable increased resistance has been observed over the past couple of years [44]. Studies have also described the transfer, by conjugation, of enterococcal and streptococcal plasmids and transposons carrying antibiotic resistance genes to *Listeria* from closely related bacteria such as *Enterococcus*, *Streptococcus* and *Staphylococcus* spp. [45] and between species of *Listeria* [46]*. L. monocytogenes* may acquire or transfer antibiotic resistance gene from mobile genetic elements such as self-transferable and mobilizable plasmids and conjugative transposons or mutational events in chromosomal genes [47]. High sensitivity levels of *Listeria* spp. towards the β-Lactams (penicillins, cephalothins) and penicillins have been reported in literature, and these antibiotics are therefore considered as the main treatment drug for listeriosis [33, 48–51]. In contrast, results obtained in this study revealed high resistance levels towards the β-Lactams: penicillin (100 %), cephalothin (60.26 %) and ampicillin (83.33 %). Arslan and Ozdemir [7] also reported resistance against ampicillin (2.1 %) and penicillin (12.8 %) in strains of *Listeria* spp. isolated from white cheese.

It has been widely reported that conventional wastewater treatment plants are unable to effectively remove antimicrobials such as antibiotics as well as a number of other chemicals from wastewater, thereby increasing the chances of bacterial pathogens resident in such wastewater effluent to acquire resistance to commonly used antibiotics due to selective pressures [52–54]. Medical and pharmaceutical discharge from hospitals has largely contributed to the increase in antibiotic concentration and therefore has led to the rise of highly resistant bacterial populations [55]. Wastewater treatment plants have therefore been considered as a rich reservoir of antibiotic and multidrug resistant organisms since the antibiotics ingested by humans are not completely processed by the body. Some of these antibiotics are expelled as waste and wind up at wastewater treatment plants [43, 56]. Rivers contaminated with urban and agricultural effluent have shown to have greater antibiotic resistant bacterial populations than areas upstream of the contamination source [57]. Antibiotic resistance in streams is also indirectly selected for by an increase in industrial wastes containing heavy metals, which could probably explain the findings of this study since both wastewater treatment plants investigated are surrounded by industrial areas, receiving wastes containing carcinogenic heavy metals and toxic chemicals as well hospital effluents. Recent findings suggest an increase in the level of multi-antibiotic resistance over the last few years [33, 58–60]. It is therefore not surprising that a high prevalence of multi-antimicrobial resistance was observed among the *Listeria* spp. in this study.

The infection or pathogenicity process of *Aeromonas* spp. is very complex and is said to involve different virulent and pathogenicity factors which either act together or separately at different stages of infection. Aerolysin gene is responsible for most of the haemolytic, cytotoxic and enterotoxic activities, which play a vital role in the initial stages of the host infection process [36, 61]. This gene was detected in 52 % of the isolates, similar to the findings of Igbinosa and Okoh [36] who reported a high presence (43 %) of the *aer* gene in *Aeromonas* spp. isolated from water samples in South Africa. Similarly, Soler et al. [62] detected this gene in 26 % of the tested environmental *Aeromonas* isolates. The presence of this *aer* gene in 52 % of the *Aeromonas* spp. obtained from treated wastewater effluent and receiving river water indicates that the isolates investigated in this study are potentially pathogenic and virulent strains of either *A. hydrophila, A. caviae and A. veroni*, where this gene is commonly found [18]. High numbers of virulence gene - containing *Aeromonas* spp. have been observed in multiple studies, involving environmental samples, with reports from Brazil, India, Italy and Spain alike [63–65]. The *lip* gene which primarily plays an integrated and coherent role in pathogenicity of *Aeromonas* spp. was detected in 68 % of the isolates. This gene is responsible for altering the host’s plasma membranes, thus increasing the severity of the infection [66–69].

In this study, some of the tested *Listeria* species were found to harbour the *act*A, *plc*A and *iap* genes, classifying them as possibly *L. monocytogenes, L. ivanovii* and *L. seeligeri*, species known to harbour these genes [35]. The haemolytic activity demonstrated by these *Aeromonas* spp. on human red blood cells is indicative of the production of the haemolysin virulence factor. Several authors have suggested that this virulence determinant is usually associated with strains of *A. hydrophila* and *A. sobria* [70–72], which are potential human pathogens. The link between haemolytic activity and enterotoxigenicity observed in this study has been well documented [71, 72]. Results obtained in this study indicate a possible presence of virulent *Listeria* and *Aeromonas* spp, which could be detrimental to the users of the receiving rivers upon exposure.

## Conclusion

The high prevalence of multi-antimicrobial resistant *Aeromonas* and *Listeria* spp. harbouring resistance genes obtained in this study is indicative of the severity of the threat these pathogens might pose to the health of the environment and other organisms exposed to the contaminated waters. Apart from the two reported emerging bacterial pathogens (*Aeromonas* and *Listeria* spp), other common bacterial indicators of water pollution, viz., *E. coli*, total coliforms, faecal coliforms, faecal *Streptococci*, *Salmonella* spp, *Shigella* spp, and *Vibrio* spp as well as other emerging bacterial pathogens including *Yersinia* spp , *Legionella* spp., *Pseudomonas* spp. were also detected in the treated effluent of these plants (Results not shown). This further confirmed previous report indicating a low reduction of microbes by treatment plants resulting in poor effluent quality with load of infectious microorganisms [73]. This is of great concern as most of the surface water samples were collected from locations which were easily accessible to animals and human populations residing in informal settlements along the river. Multidrug resistant organisms found in this study were also resistant to some of the commonly used antibiotics and this is particularly worrisome in a province with a high number of immunocompromised individuals due to the high HIV and TB pandemic as this will impact on treatment regime. Findings from this study further highlight the need for the Department of Water Affairs to revise the current guidelines and standards to include the emerging bacterial pathogens, which are often detected even in the absence of commonly used indicator organisms. There is also need for constant evaluation of the wastewater treatment plants to ensure efficiency and compliance to set guidelines in order to protect public and environmental health.

## Abbreviations

*aer*: Aerolysin
ARI: Antibiotic resistance index
*gyrB*: Gyrase B
*hlyA*: Hemolysin
*lip*: Lipase
*actA*: Actin assembly-inducing protein
*iap*: p60 invasion proteins
*plcA*: Phospholipase
MAR: Multiple antibiotic resistance
PCR: Polymerase chain reaction
